# MeRIP-Seq initially revealed the role of m6A modification in Chinese sacbrood virus-infected *Apis cerana* larvae

**DOI:** 10.3389/fmicb.2025.1563240

**Published:** 2025-04-30

**Authors:** Yuming Liu, Hua Bai, Huitong Qiu, Dongliang Fei, Mingxiao Ma

**Affiliations:** College of Animal Husbandry and Veterinary, Jinzhou Medical University, Jinzhou, China

**Keywords:** Chinese sacbrood virus, N6-methyladenine, *Apis cerana*, antiviral gene, *AcMETTL3*

## Abstract

Chinese sacbrood virus (CSBV) is highly lethal to honeybee larvae (especially the larva of *Apis cerana*) and causes considerable losses to beekeeping industry. N6-methyladenine (m6A) modification of mRNA is a predominant post-transcriptional modification in eukaryotes and plays a role in viral infection. However, the role of m6A modification in CSBV infection remains unclear. Herein, we performed high-throughput sequencing for m6A-seq in CSBV-infected and non-infected larvae to investigate host transcriptome-wide m6A modifications and identify m6A-modified genes. A total of 671 variant peaks were identified. Combined analysis of m6A modification and mRNA expression revealed that a significant correlation between mRNA methylation modifications and expression levels observed for 668 Genes. It was proved that CSBV infection can cause important m6A modification changes in host. We examined the effects of CSBV infection on expression of two methylation regulatory genes by qPCR. At the same time, we verified the effect of two methylation regulatory genes on CSBV replication using RNAi technology. This study demonstrated for the first time that CSBV infection can cause m6A modification changes in *A. cerana* larvae, and comprehensively analyzed the m6A modification pattern of its mRNA, and CSBV infection significantly promoted the expression of *AcMETTL3* (Ac represents *A. cerana*, *p* = 0.007), but had no effect on the expression of *AcMETTL14*. It was further confirmed that *AcMETTL3* had a significant negative regulatory effect on CSBV replication (*p* = 0.0432). These results lay a foundation for further exploration of the role of m6A modification in CSBV infection.

## Introduction

Honeybees are major crop pollinators worldwide and model organisms for the study of development, behavior, and learning (Leonard et al., 2020). Honeybee populations have been declining due to various biological and environmental factors. Among these, the sacbrood virus (SBV), first isolated from honeybee colonies in 1982, significantly impacts honeybee health and lifespan (Potts et al., 2010). Along the same lines, Chinese sacbrood disease (CSBD) is a widespread, highly prevalent and rapidly spreading infectious disease caused by CSBV. While CSBV/SBV infection leads to larval mortality, adult bees typically show no overt clinical symptoms. However, infected adult bees exhibit significant physiological impairments, including: reduced lifespan, decreased nectar foraging capacity, impaired flight performance (Li et al., 2019). However, research on the pathogenesis of CSBV remains relatively limited, which has significantly hindered the development of novel antiviral drugs targeting viral invasion. As a result, effective control measures for CSBV are still lacking.

The reversible N6-methyladenosine (m6A) modification, one of numerous RNA modifications, serves as an important regulator of post-transcriptional gene expression in eukaryotes (Huang et al., 2018). This modification is dynamically regulated: installed cotranscriptionally by the m6A methyltransferase complex, removed by m6A demethylases, and recognized by m6A-binding proteins (Meyer and Jaffrey, 2017). m6A regulates RNA processing and metabolism, including mRNA splicing, nuclear export, RNA stability, and translation, via the recognition of m6A binding proteins (Roignant and Soller, 2017). Ultimately, m6A modification affects various biological processes, such as meiosis (Ivanova et al., 2017), stem cell properties (Xie et al., 2024), and neurodevelopment (Li et al., 2018).

METTL3 (methyltransferase-like 3), the core catalytic component of the m6A methyltransferase complex, partners with METTL14 to mediate adenosine methylation on mRNA molecules. Studies have shown that in insects, METTL3 and METTL14 jointly mediate RNA m6A modification and influence male reproductive development in *Bactrocera dorsalis* by regulating the synthesis of 20-hydroxyecdysone (Zhang et al., 2025). Additionally, METTL3 is evolutionarily conserved in insects. Research indicates that knocking down *METTL3* in *Bombyx mori* (silkworms) affects cellular redox enzyme activity, amino acid biosynthesis, and other metabolic processes, while also significantly impacting the Wnt and Toll/IMD signaling pathways (Lin et al., 2023). However, the biological functions of METTL3 in bee species require further investigation.

There are numerous studies on the impact of m6A modification on mammalian viral infections. For example, EV71 promotes SUMO and ubiquitination modifications of 3D through METTL3 to promote viral replication (Hao et al., 2019). However, research on the relationship between m6A modification and insect-virus interactions remains relatively limited. A study demonstrated that infection with rice stripe virus (RSV) in the small brown planthopper (SBPH, *Laodelphax striatellus*) led to the downregulation of *LsIMPDH* expression. This suppression was mediated through m6A modification regulated by *LsMETTL3* and *LsYTHDF3*, resulting in reduced GTP levels and ultimately inhibiting viral replication (Zhu et al., 2024). For honeybees, existing studies have demonstrated a connection between m6A modification and their growth and development. m6A modifications are reported to play a key role in honeybee larval development and hierarchical differentiation (Wang et al., 2021). It also plays an important role in the study of bee aggressive behavior and in exploring the effects of fipronil (a phenylpyrazole insecticide) on honeybee larvae and adults (Bresnahan et al., 2023; Fan et al., 2023). Recently, Bataglia et al. (2021, 2022) have laid the foundation for study of RNA methylation in honey bees by investigating the active gene mechanisms of epigenetic RNA modification and transcriptional expression of RNA methyltransferases in different parts of worker bees. However, the effects of honeybee viral infections on host m6A modifications, as well as how m6A modifications influence viral replication, remain unexplored areas of research.

To explore m6A’s role in honeybee CSBV infection, this study characterizes m6A modification patterns in *Apis cerana* worker larvae. The 3-day-old larvae were picked to laboratory and incubated for 1 day, after removing the dead larvae, virus infection was performed (4-day-old larvae), and larval samples were collected after 48 h of incubation (when the larvae reached the age of 6 days) for m6A modification histology analysis and experimental assays. m6A modification was significantly increased during CSBV infection of larvae compared with that in the uninfected control group, and gene expression was negatively correlated with m6A methylation (Student’s *t*-test). Further analysis revealed that CSBV infection stimulated the expression of multiple methylation-modifying enzymes in the *A. cerana* larvae. Thus, m6A modification may have an important regulatory role in CSBV infection in honeybees.

## Materials and methods

### Viruses and viral infection

Based on our preliminary findings confirming the high susceptibility of 4-day-old larvae to CSBV infection and observing significant viral load increases at 48 h post-infection (hpi), we established standardized infection protocols and detection criteria (Bai et al., 2025).

To purify CSBV, we collected 109 *A. cerana* larvae aged 3–4 days showing obvious disease symptoms. The larvae were homogenized in 5 mL of sterile phosphate-buffered saline (PBS) using a tissue grinder. The homogenate was centrifuged at 8,000 rpm for 30 min at 4°C, and the supernatant was filtered twice through a 0.22 μm membrane. The absence of other viral infections was confirmed by RT-PCR. The collected viral solution was further purified by cesium chloride (CsCl) density gradient centrifugation, followed by dialysis to remove CsCl. The purified virus was confirmed to be free of other bee viruses by PCR. The viral copy number was quantified using total RNA as the template. cDNA was synthesized using a reverse transcription kit (Takara), followed by qPCR amplification with TransStart Probe qPCR SuperMix. With thermal cycling conditions including 94°C denaturation for 30 s, followed by amplification at 94°C for 5 s, and 59°C for 30 s for 40 cycles. RNA-free water was used as the template for negative control reactions, with each sample technically replicated three times on the same plate. The primers and probes used in the PCRs are indicated in Table 1. The viral titer was determined to be 1 × 10^7^ copies/μl. The purified virus solution was stored at-80°C for use.

**Table 1:** Primer sequence of qPCR.

Gene	Forward primer sequence	Reverse primer sequence
*AcMETTL3*	GCGACTAGTCACAAGCCTGA	CTCGTTCCCGGACTCATACG
*AcMETTL14*	TGTGGTGACCAGTGATGATGAA	TACCACTTTTACCACCTGCCC
*β-actin*	ATGCCAACACTGTCCTTTCTGG	GACCCACCAATCCATACGGA
CSBV	CCTGGGAAGTTTGCTAGTATTTACG	CCTATCACATCCATCTGGGTCAG
CSBV-Probe	5′-(FAM) CGACATACCCGCAAATTCAGCACGC (Eclipse)-3’	

For consistent viral dosing, we developed a controlled infection protocol where viral solution was combined with larval food (sugar water) in measured 6 mL quantities, with careful documentation of initial mixture weights. The larvae were transferred into the cup for viral exposure. Following a 6.5-h incubation period, they were carefully removed while maximizing liquid retention. The remaining larval solution was returned to the cup, and the post-infection weight was recorded. The amount of virus ingested by the larvae was determined by subtracting the first weight from the second weight. In subsequent experimental analyses, samples with abnormal viral doses were excluded, ensuring that each larva ingested an equivalent amount of virus while maintaining consistency with the natural infection process of CSBV.

### Sample collection for m6A-seq

**Figure 1:**
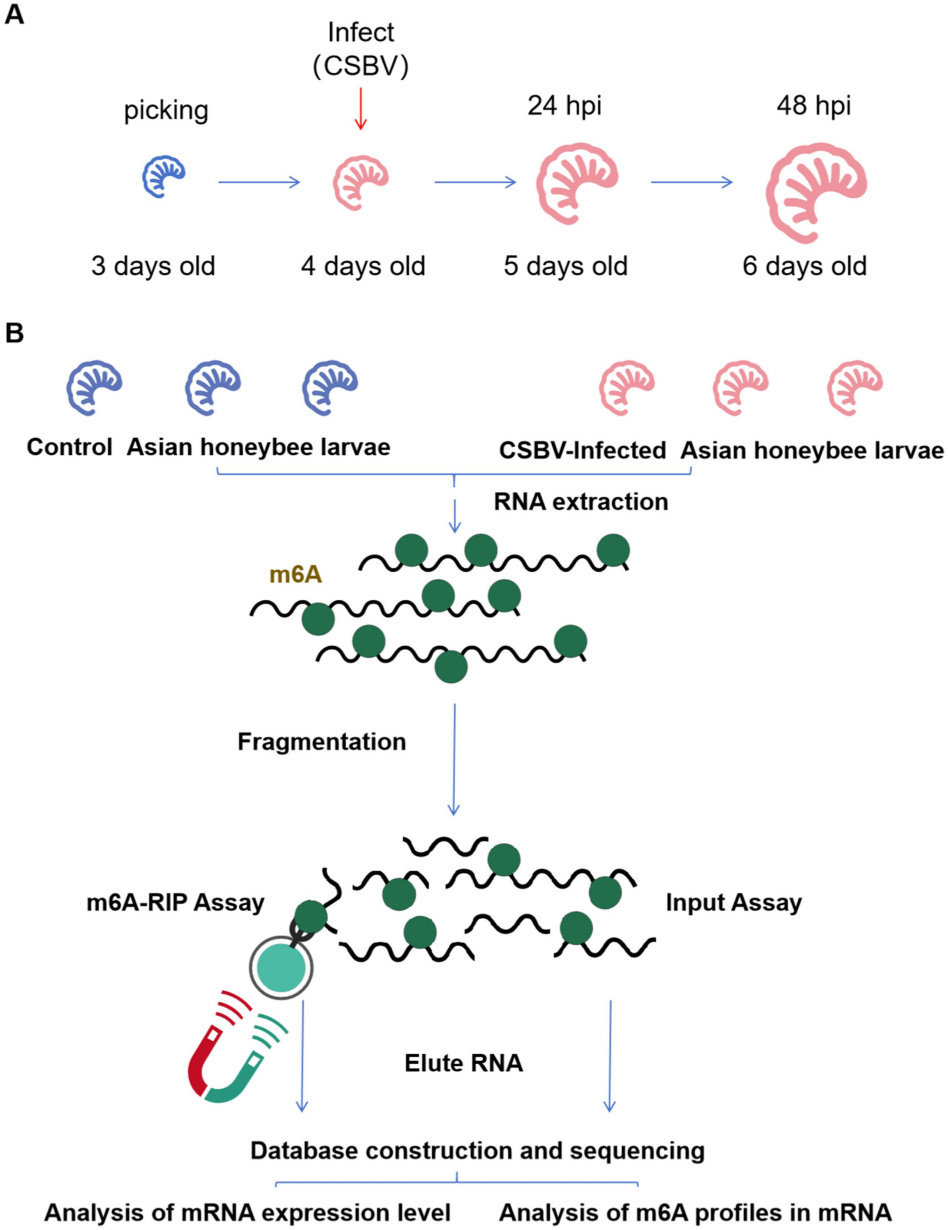
Infection scheme and MeRIP-Seq flow. **(A)** Samples were collected after CSBV infected for sequencing. **(B)** Summary of the MeRIP-Seq process.

By restricting egg-laying in healthy *A. cerana* queens, we obtained honeycomb frames densely populated with 4-day-old *A. cerana* larvae. Next, we performed PCR and RT-qPCR assays to screen the selected larvae for common honeybee bacterial and viral diseases (e.g., *Melissococcus plutonius*, Deformed wing virus, etc.). The results confirmed that this colony was healthy and disease-free. Larvae that were injured during collection or exhibited abnormal morphology, coloration, or size compared to normal 4-day-old larvae were discarded prior to viral infection. 4-days-old *A. cerana* larvae were selected for infection, and larvae samples for detection were collected 48 h after infection (Figure 1A). Treatment groups included control and infected groups that were each divided into three replicates, with 10 larvae in each replicate. The collected larvae samples were snap-frozen in liquid nitrogen and sent to gene sequencing company (E-GENE, Shenzhen) for m6A-Seq (Figure 1B).

### RNA extraction and m6A MeRIP

Total RNA was extracted using Trizol (Invitrogen), and the concentration and integrity of extracted RNA measured using a Qubit RNA HS Assay and Agilent 2,100 Bioanalyzer (Agilent Technology), respectively. For the MeRIP experiment, approximately 20 μg of total RNA from each sample was fragmented using 10X RNA Fragmentation Buffer (Invitrogen) by incubating in a preheated thermal cycler for 10 min at 70°C. Fragmented RNA was pelleted by ethanol precipitation. Protein A and protein G magnetic beads were washed twice with IP buffer (150 mM NaCl, 10 mM Tris–HCl pH 7.5, 0.1% IGEPAL CA-630 in nuclease-free H_2_O) before incubating with 5 μg m6A antibody (Millipore) at 4°C for 2 h. After two washes with IP buffer, the antibody-bead complexes were resuspended in 500 μL of the IP reaction mixture including fragmented total RNA and incubated for 4 h at 4°C. To validate the specificity of the antibody, we performed immunoprecipitation (IP) using synthetic RNA fragments containing known m6A modifications to confirm effective enrichment. In parallel, we conducted IP with unmodified RNA sequences of identical composition to verify the absence of non-specific binding (antibody enrichment efficiency >5-fold, negative control signal <10%). These validation results ensure the reliability of the subsequent sequencing data. The immunoprecipitated m6A RNA with protein A/G magnetic beads was then washed three times using IP buffer for 10 min each at 4°C. Finally, the bead complexes were resuspended in 100 μL of m6A competitive elution buffer with continuous shaking for 1 h at 4°C. The supernatant containing the eluted m6A RNA was collected and purified using phenol: chloroform: isoamyl alcohol (125:24:1).

### m6A-seq assay and data analyses

MeRIP libraries using the eluted RNA were constructed using SMARTer Stranded Total RNA-Seq Kit version 2 (Takara/Clontech) according to the manufacturer’s protocol. All libraries were analyzed using an Agilent 2,100 Bioanalyzer (Agilent Technologies) and quantified via real time PCR before sequencing. The raw sequencing data were subjected to quality control steps, including adapter removal and low-quality base trimming, using the Trimmomatic software with the following parameters: SLIDINGWINDOW:30:15, AVGQUAL:15, LEADING:15, TRAILING:15, and MINLEN:30. Clean reads were mapped to the genome assembly of *A. mellifera* (Amel_4.5) using HISAT (version 2.1.0) with default parameters. The sequencing depth was as follows: Immunoprecipitation (IP) group: Average depth of 28.27×, Input control group: Average depth of 36.83×. The alignment file (SAM) was transformed to a BAM file and filtered using the following steps: (1) only unique properly aligned reads were kept; (2) reads with low MAPQ (<30) were removed; and (3) reads mapped to blacklist regions were removed. R package exomePeak (version: 2.1.2) was used to call peaks and detect differential peaks from filtered alignment files with the following parameters: “WINDOW_WIDTH = 200 SLIDING_STEP = 30 FRAGMENT_LENGTH = 150 DIFF_PEAK_ABS_FOLD_CHANGE = 2 FOLD_ENRICHMENT = 2.” The annotation file used to annotate the peaks was downloaded from UCSC. Only peaks with an FDR of <0.05 and foldchange of ≥2 were identified as being significantly differential. Gene expression calculation was performed using StringTie software with default parameters. Gene expression profiling was based on the number of reads. Transcripts per million mapped read values were used to estimate the expressed values and transcript levels. DESeq2 was selected to identify differentially expressed genes (DEGs). Genes with an adjusted *p*-value (padj) of <0.05 and abs [log2(fold change)] of >1 were considered as DEGs. For the correlation analysis of metagene profiling/plots we applied the metagene2 software.^1^

### Analysis of m6A-enriched region-associated gene enrichment

Hypergeometric testing was performed for significance enrichment analysis, with a statistical threshold set at FDR < 0.05 and Benjamini-Hochberg correction applied for multiple testing. A minimum of ≥5 genes per functional term/pathway was required to ensure analysis reliability, while the reference background gene set (either all detected genes or differentially expressed genes) was explicitly defined. All analyses used a fold enrichment threshold > 2 to ensure result quality, with key functions and pathways displayed via TopN presentation.

Gene Ontology (GO) is an international standard classification system for gene function, which provides a set of dynamically updated standardized vocabularies to describe the properties of genes and gene products in organisms, which can be mined for a number of biologically relevant pathways. GO is categorized into three Ontologies, namely: Molecular Function (MF), Cellular Component (CC) and Biological Process (BP).

The KEGG is a simulation of biological systems, including molecular wiring diagrams of interactions, reactions, and networks of relationships consisting of molecular structural units of genes, proteins, and compounds, as well as information on diseases and drugs.

Gene Ontology and KEGG enrichment analysis was done with m6A modification-associated genes. This analysis helps to understand which biological functions m6A-modified genes are mainly involved in. The Gene Ontology (GO) and pathway analysis of the coding genes with differential methylation or expression levels were performed by GO^2^ and the Kyoto Encyclopedia of Genes and Genomes (KEGG).^3^ The m6A abundance on the genome was visualized with the interactive analysis tool Integrative Genomics Viewer (IGV) software.^4^

### qPCR validation for m6A modification genes

qPCR was performed for two selected genes (*AcMETTL3* and *AcMETTL14*) using PowerUp SYBR Green Master Mix (A25742, Thermo Fisher Scientific) on a QuantStudio 1 system (Applied Biosystems) and normalized with an external reference sequence (actin related protein 1 gene, Gene ID: LOC108003298). The PCR process was divided into two steps, starting with denaturation at 95°C for 15 s and annealing and extension at 60°C for 1 min for a total of 40 cycles. The relative amount of the target gene was calculated by normalizing the internal reference gene *β-actin* and analyzed by threshold cycling (2^−ΔΔCt^ method). Primers for qPCR analysis are listed in Table 1.

### Detection of CSBV replication levels

For each experimental group, 6–8 larvae were collected and processed for total RNA extraction using TRIzol reagent (TransGen Biotech, ET111-01-V2) according to the manufacturer’s specifications. RNA concentration and purity were quantitatively assessed using a NanoDrop 2000 spectrophotometer (Thermo Fisher Scientific, United States). First-strand cDNA synthesis was performed employing the RevertAid First Strand cDNA Synthesis System (Thermo Fisher Scientific, United States). Subsequent quantitative PCR amplification was conducted using a QuantStudio 3 Real-Time PCR System (Thermo Fisher Scientific, United States), with thermal cycling conditions including 94°C denaturation for 30 s, followed by amplification at 94°C for 5 s, and 59°C for 30 s for 40 cycles. RNA-free water was used as the template for negative control reactions, with each sample technically replicated three times on the same plate. The primers and probes used in the PCRs are indicated in Table 1.

### Synthesis and delivery of dsRNA

Double-stranded RNA of *AcMETTL3* and *AcMETTL14* genes of *A. cerana* were designed and synthesized (sequences are shown in Table 2). To feed dsRNA (5 μg/μL) to honey bee larvae, the synthesized dsRNA stock solution was dissolved in RNase-free water at a ratio of 1:100 to prepare the working solution. The working solution was mixed with larval feed and each larva was fed 100 μL. For the experimental group fed with *dsAcMETTL3/dsAcMETTL14*, we established a control group fed with *dsGFP* (with 10 larvae pooled per sample and 3 biological replicates per group to ensure experimental reproducibility). At multiple consecutive time points post dsRNA feeding (e.g., 24, 60, and 96 h), we assessed the knockdown efficiency of target genes. Comparison with the *dsGFP* group confirmed successful suppression of target genes, thereby excluding potential off-target effects of dsRNA (ineffective knockdown or unintended suppression of non-target genes). Finally, while examining the impact of target genes on viral replication, we simultaneously verified target gene expression at each sample collection time point to ensure the accuracy of our research findings.

**Table 2:** The sequence of dsRNA.

dsRNA sequence	dsForward	dsReverse
*dsMETTL14*	TAATACGACTCACTATAGGGCACTTTTGTCTTGGTAGGCG	TAATACGACTCACTATAGGGTTTGGTAACGCGGTGTATTG
*dsMETTL3*	TAATACGACTCACTATAGGGCCTGCTCTTCAAGATGAAGG	TAATACGACTCACTATAGGGCATTGAGTTTCCATCCGGAT
*dsGFP*	TAATACGACTCACTATAGGGGCGAGGGCGATGCCACCTAC	TAATACGACTCACTATAGGGCACGCTGCCGTCCTCGATGT

### Statistical analyses

To mitigate biological bias, larvae were randomly distributed into each group. All data points were biological and not technical replicates (*n* = 3 ~ 8). Data are shown as means ± standard deviation (SD), and their significant differences were analyzed and compared in the GraphPad Prism 9 program (MDF Co. Ltd.) using either Student’s *t*-tests (*p < 0.05, **p < 0.01, ***p < 0.001; ^NS^*P* > 0.05, not significant).

## Results

### m6A modification omics of larva

RNA-seq libraries were generated from input RNA and m6A-IP RNA samples derived from fragmented total RNA of CSBV-infected and control *A. cerana* larvae. MeRIP-seq produced 6,244,116,300–17,458,354,500 raw reads and 5,535,203,849–15,473,949,018 clean reads from input or IP RNA from control and CSBV-infected *A. cerana* larvae, and 41,594,264–116,284,196 high-quality reads were matched to the acer genome (as shown in Table 3). The m6A-seq dataset is similar to the RNA-seq dataset, and both of them can be mined for potential functional genes with significant differences in expression changes. The gene that hosts said m6A sites with fold changes ≥ 2 were defined as valid sites. A total of 2,675 expressed mRNA transcripts were identified in CSBV-infected and control *A. cerana* larvae. Following the mapping of methylated RNA segments to the transcriptome, the data we get in each group are: 1,543–7,512 and 4,629–7,175 m6A peaks were identified in control and CSBV-infected *A. cerana* larvae, respectively. Based on these data, we determined that methylated transcripts contained an average of 0.57–2.81 m6A peaks in CSBV-infected *A. cerana* larvae, compared to 1.73–2.68 peaks in control larvae (as shown in Table 4).

**Table 3:** Comparison and filtered data information.

SampleID	RawReadNum	RawBaseNum	CleanReadNum	CleanBaseNum	CleanRate
Control_1_IP	45,656,452	6,848,467,800	45,566,924	6,208,999,827	90.66%
Control_1_Input	41,952,760	6,292,914,000	41,866,948	5,718,730,917	90.88%
Control_2_IP	44,617,912	6,692,686,800	44,580,574	6,072,503,320	90.73%
Control_2_Input	116,389,030	17,458,354,500	116,284,196	15,473,949,018	88.63%
Control_3_IP	50,753,376	7,613,006,400	50,719,730	6,790,096,659	89.19%
Control_3_Input	45,939,814	6,890,972,100	45,902,736	6,026,853,847	87.46%
CSBV_1_IP	48,070,502	7,210,575,300	47,963,444	6,626,110,543	91.89%
CSBV_1_Input	49,149,892	7,372,483,800	49,049,316	6,765,928,606	91.77%
CSBV_2_IP	52,149,296	7,822,394,400	52,119,044	7,102,018,516	90.79%
CSBV_2_Input	41,627,442	6,244,116,300	41,594,264	5,535,203,849	88.65%
CSBV_3_IP	44,437,766	6,665,664,900	44,412,072	6,095,405,393	91.44%
CSBV_3_Input	52,130,112	7,819,516,800	52,078,054	6,913,159,816	88.41%

**Table 4:** The number, total length and average length of peak were counted.

Sample	nPeak	Total_Length	Mean_Length
Control_1	1,543	1,013,978	657.15
Control_2	7,512	7,639,217	1016.94
Control_3	6,812	7,426,062	1090.14
CSBV_1	4,629	4,558,488	984.77
CSBV_2	7,175	9,889,309	1378.3
CSBV_3	6,706	8,025,000	1196.69

### Peak distribution of m6A in mRNA transcripts

**Figure 2:**
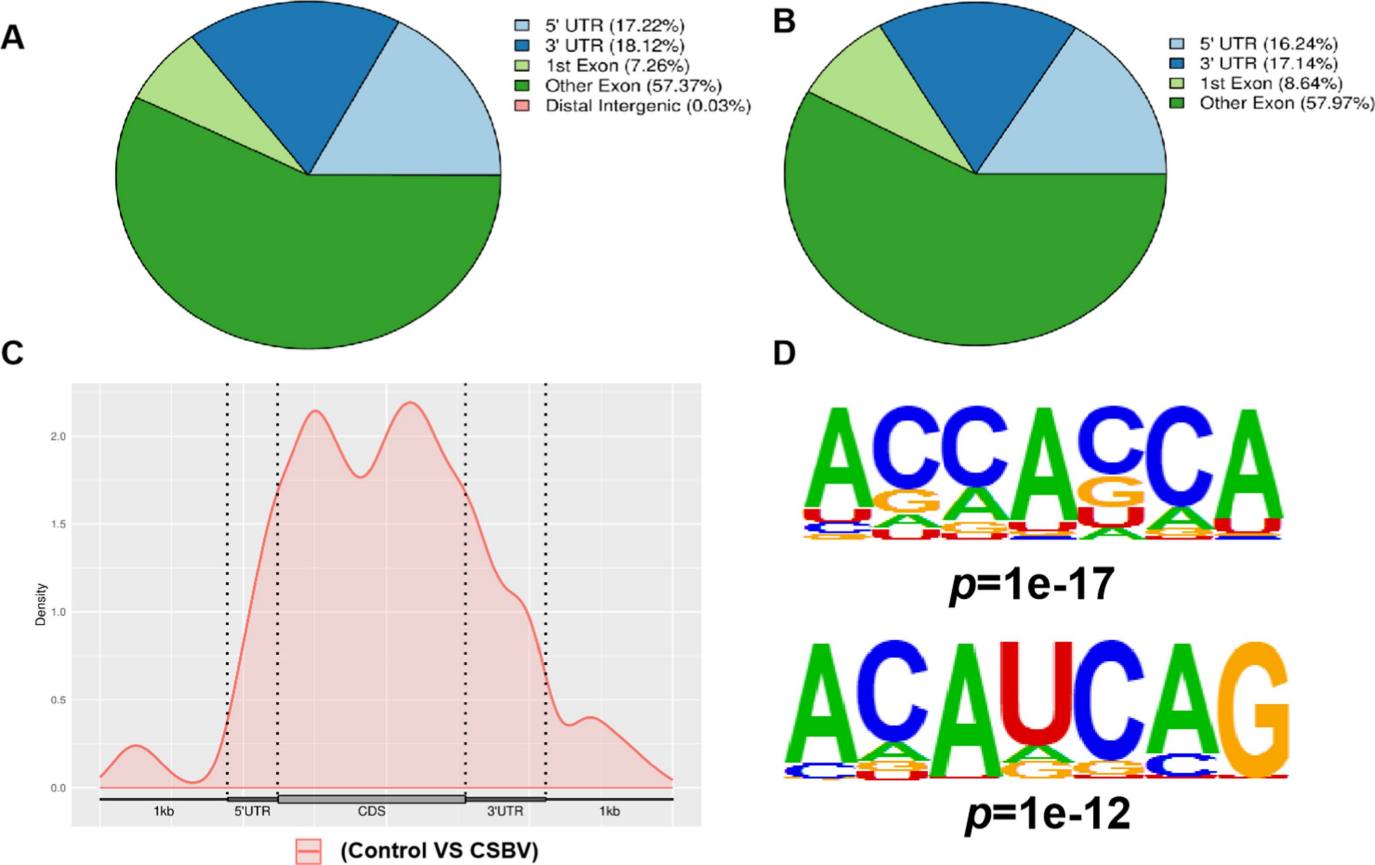
Peak distribution of m6A in mRNA transcripts. Pie charts illustrating the percentage of m6A peaks in the individual region of mRNA transcripts from **(A)** control and **(B)** CSBV-infected *Apis cerana* larvae. **(C)** Metagene plots displaying the abundance of m6A peaks along mRNA transcripts. **(D)** Base sequences of m6A modification motifs in control and CSBV-infected *Apis cerana* larvae.

Our analysis of m6A modification distribution revealed their predominant localization within coding sequences (CDS), with comparatively lower abundance in both 3′ and 5′ untranslated regions (UTRs). After CSBV infection, the changes in the localization of m6A modification in other exons and 3′-UTRs were more pronounced (Figures 2A,B). Metagene profiling was performed to determine the distribution patterns of m6A peaks in the structure of mRNAs and showed that the peaks were primarily enriched in the CDS (Figure 2C). We then investigated the motifs distributed in the region near the m6A peaks based on their *E*-value. The base sequences were ACCACCA and ACAUCAG (Figure 2D), which differed from the conventional DRACH/RRACH conserved motif.

### Analysis of different m6A peaks and genes post-CSBV infection

**Figure 3:**
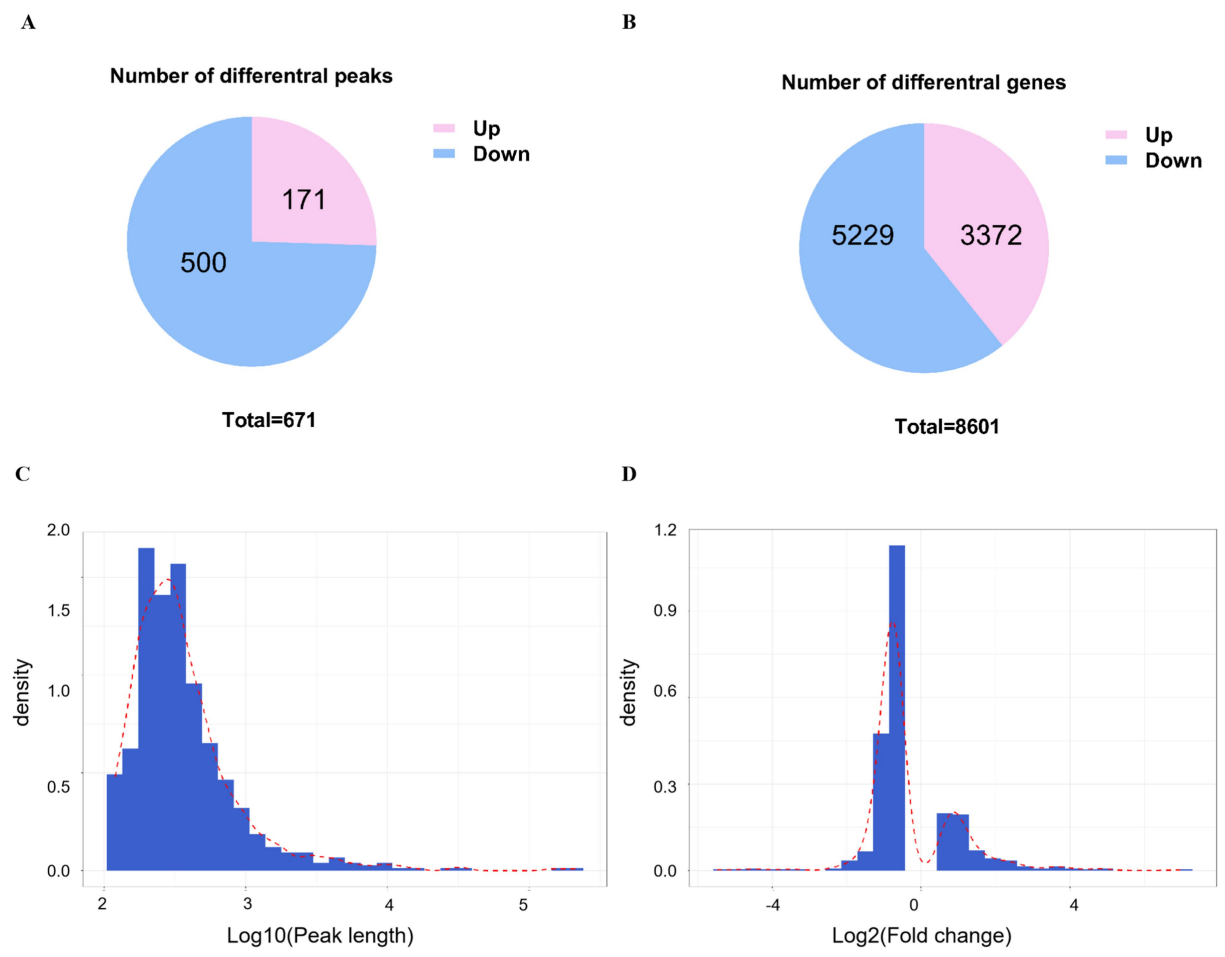
Analysis of different peaks and genes post-CSBV infection. **(A)** The number of substantially changed m6A peaks and **(B)** their distribution among genes. **(C)** The distribution of peak length and **(D)** fold change of all peaks.

To identify differences in m6A modification and gene expression, we analyzed methylation levels within m6A peaks and corresponding mRNA in infected versus control samples. A total of 671 differential m6A peaks distributed across 8,601 genes were screened, of which 500 peaks distributed across 5,229 genes and 171 peaks distributed across 3,372 genes were substantially changed (Figures 3A,B). The distribution of peak length and fold change among differential m6A peaks are shown in Figures 3C,D.

### GO and KEGG analysis of differential m6A modified genes

**Figure 4:**
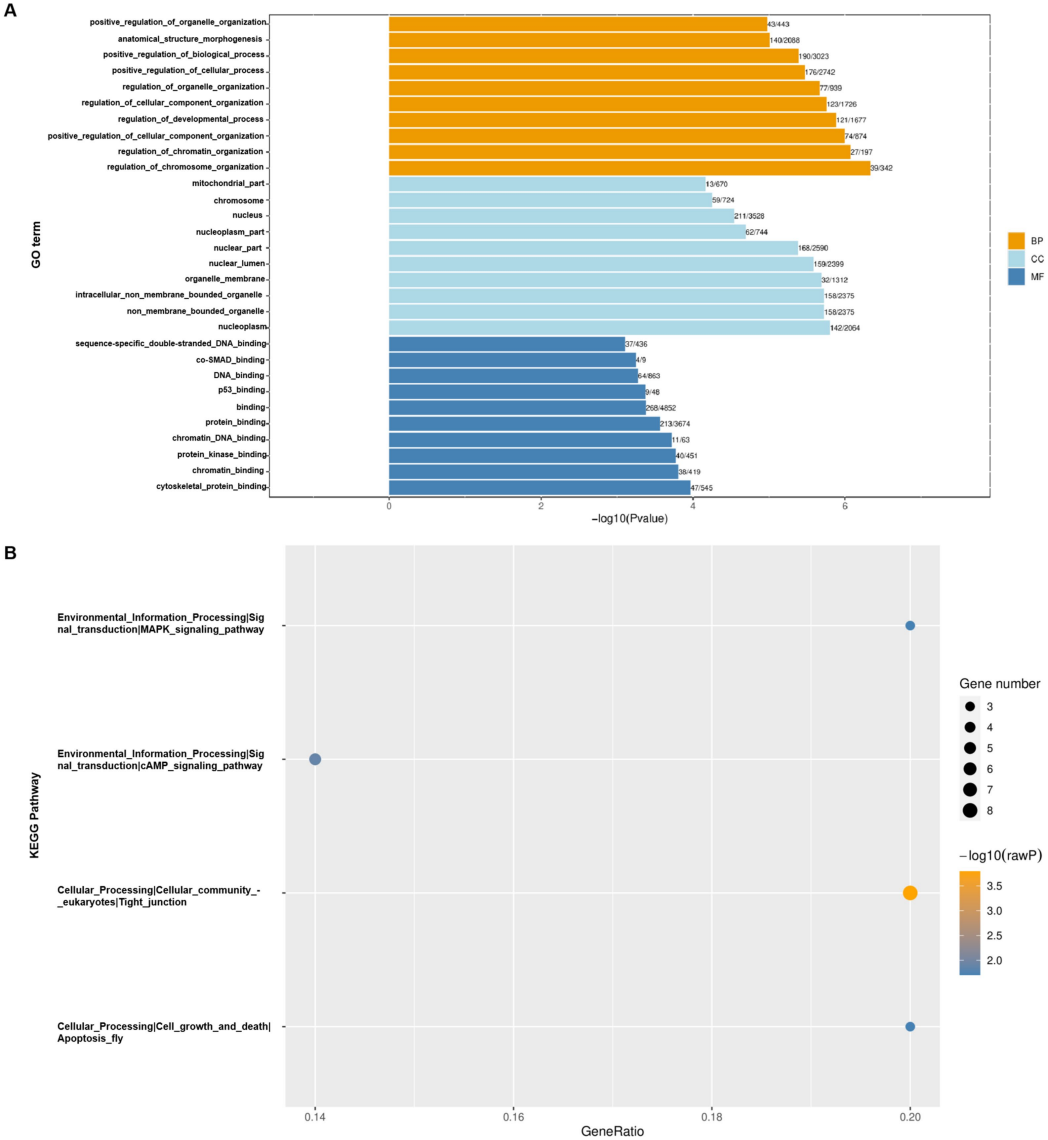
GO **(A)** and KEGG **(B)** pathway analysis of differentially m6A-modified genes.

We conducted GO and KEGG pathway analysis on genes with differential m6A methylation to more deeply explore the m6A modification during CSBV infection. Gene Ontology analysis showed that the top 30 GO terms with the most significant enrichment mainly involved the processes of molecular function (BP), metabolic process (MF) and cellular component (CC), with the most significant changes in molecular process (Figure 4A). This result indicates that during CSBV infection, the alterations of these biomolecules are highly likely to be an adaptive response made by the host at the molecular level in response to CSBV infection. By influencing the molecular processes, m6A modification may play a role in key links such as the regulation of the recognition and invasion of host cells by the virus, as well as subsequent viral replication and transmission. From the results of the KEGG pathway analysis, m6A modification genes are extensively involved in processes such as the MAPK signaling pathway, cAMP signaling pathway, tight junctions, and apoptosis (Figure 4B). The regulation of the MAPK signaling pathway by m6A modification genes implies that they may play an important role in the processes determining host cell stress induced by CSBV infection. The fact that m6A modification genes are extensively involved in the cAMP signaling pathway suggests that m6A modification genes may affect the normal physiological metabolism of host cells by regulating the cAMP signaling pathway, creating favorable conditions for viral infection and replication. As for the regulation of the apoptosis pathway, it is likely that m6A modification genes determine whether host cells undergo apoptosis to limit viral spread or remain viable to provide a replication site for the virus by regulating the apoptosis process.

### Transcriptome and methylome association analysis in larva

**Figure 5:**
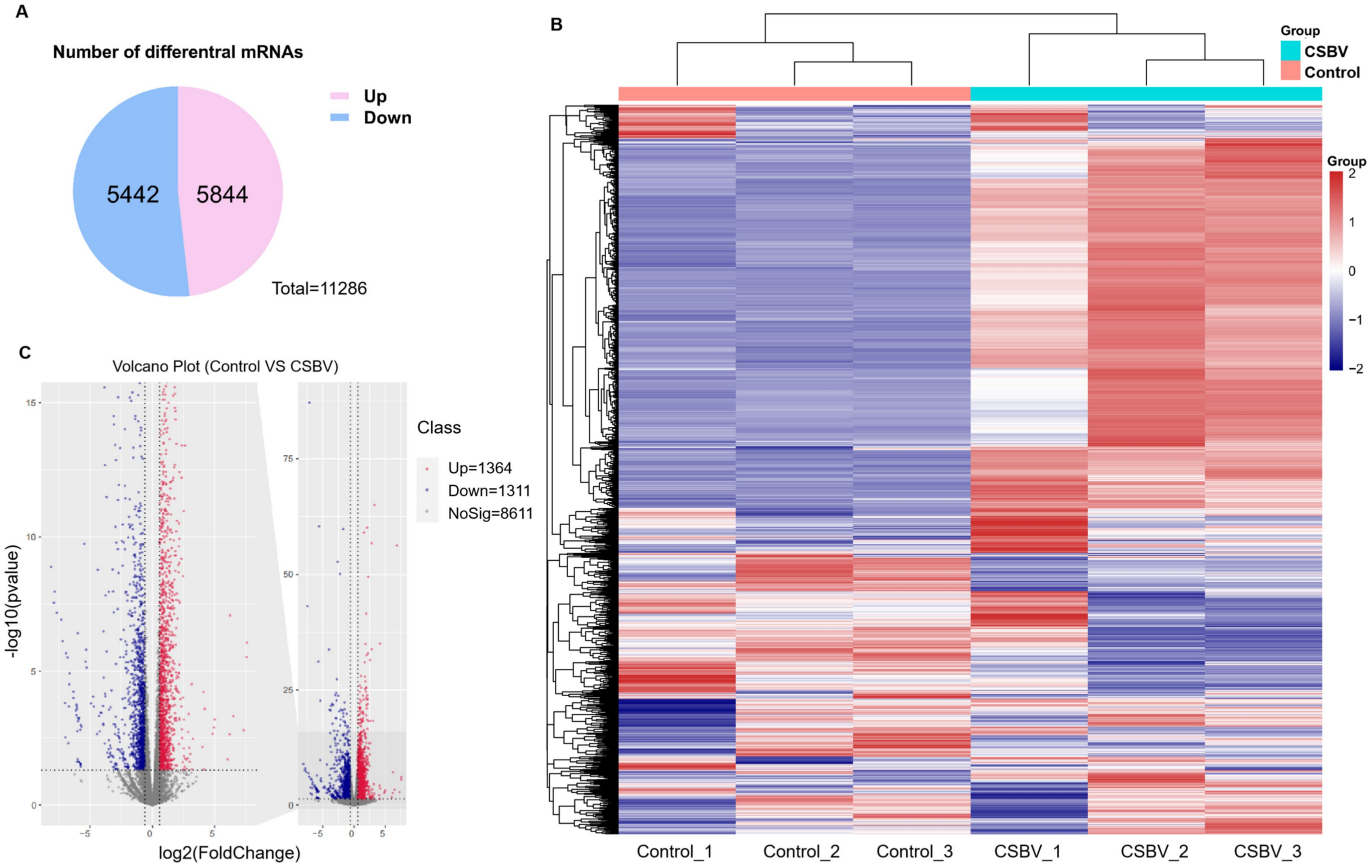
Differences in mRNA expression levels after CSBV infection. **(A)** Venn diagram showing the total number of upregulated and downregulated mRNAs. **(B)** Heatmap displaying the relative expression levels of genes in three CSBV-infected and three control samples. **(C)** Volcano plot showing mRNA with differential expression between the CSBV-infected and control samples.

We next examined differentially expressed genes between CSBV-infected and control *A. cerana* larvae to further explore how CSBV-induced m6A modifications influence gene expression. In total, the expression levels of 5,844 genes were upregulated while those of 5,442 genes were downregulated as represented by the Venn diagram (Figure 5A). The heatmap revealed significant differences in expression levels between the infected and the control groups, whereas those between the three replicate samples of the groups were similar (Figure 5B). The volcano plot represents the significant differentially expressed mRNAs post-CBSV infection (Figure 5C).

### Effect of CSBV infection on expression of m6A modified genes *AcMETTL3* and *AcMETTL14*

**Figure 6:**
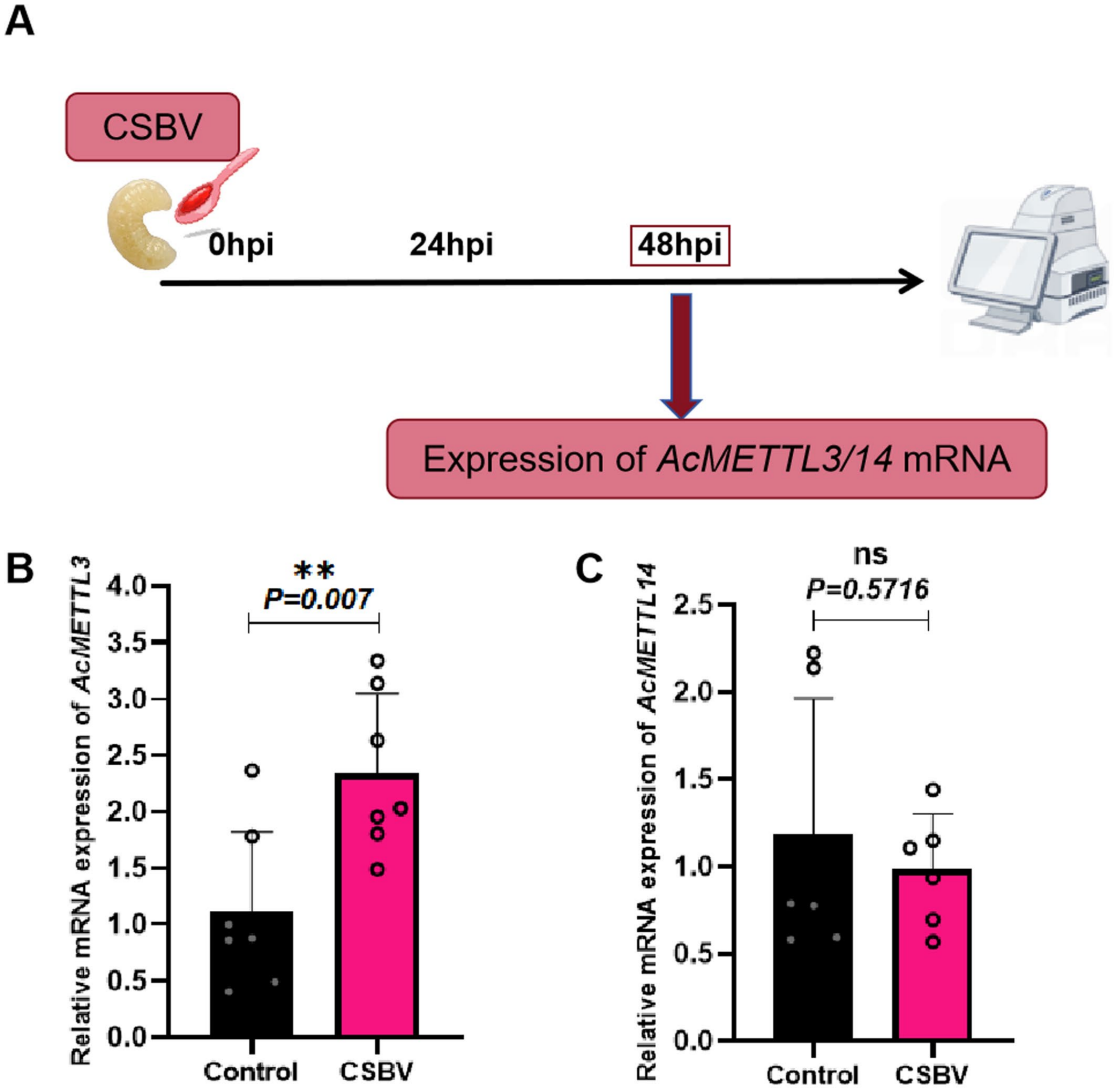
Effects of CSBV infection on the expression of m6A modified genes (*AcMETTL3* and *AcMETTL14*). **(A)** Virus infection scheme design. **(B,C)** The expression of *AcMETTL3* and *AcMETTL14* in CSBV-infected larvae was detected by qPCR (*n* = 6 ~ 8 larvae/group). Student’s *t*-test was used to compare control and CSBV groups. (**p < 0.01; *^NS^P* > 0.05, not significant). Data represented as mean ± SD.

In previous studies, we observed that the expression of AcMETTL3 and AcMETTL14 varied with the progression of CSBV infection (Bai et al., 2025). Next, to explore more new antiviral targets, we performed RT-qPCR detection of two m6A modified genes (*AcMETTL3* and *AcMETTL14*) with that displayed significant changes in expression after CSBV infection (Figure 6A). After infection, the expression of *AcMETTL14* was not significantly changed, whereas that of *AcMETTL3* was significantly increased. Therefore, *AcMETTL3* may be involved in regulation function of CSBV infection. These results will provide guidance for our future research (Figures 6B,C).

### *AcMETTL3* regulates CSBV replication

**Figure 7:**
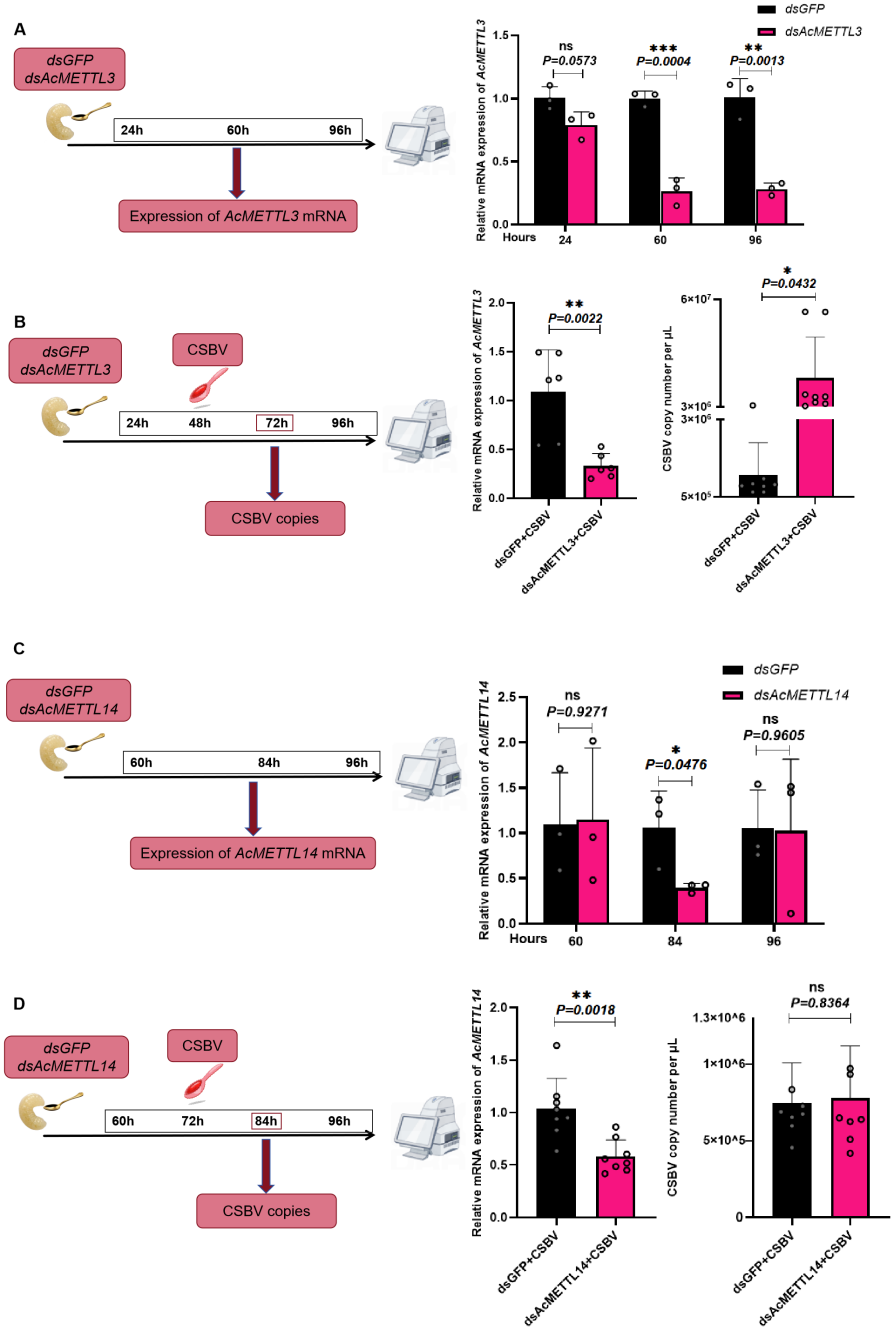
*AcMETTL3* knockdown negatively regulates the host innate immune response and promotes CSBV replication. **(A,C)** Schematic of experimental procedure, wherein expression of m6A modified genes was recorded at different time points after administering *dsMETTL3*/*dsMETTL14* or *dsGFP* through feeding in *Apis cerana* larvae (*n* = 3 larvae/group). **(B)** Relative mRNA expression levels of *AcMETTL3* and CSBV copies number in control (*dsGFP*, black) and experimental (*dsAcMETTL3*, pink) groups (*n* = 6 ~ 8 larvae/group). **(D)** Relative mRNA expression levels of *AcMETTL14* and CSBV copies number in control (*dsGFP*, black) and experimental (*dsAcMETTL14*, pink) groups (*n* = 6 ~ 8 larvae/group). Student’s *t*-test was used to compare *dsGFP* and *dsMETTL3/dsMETTL14*-treated groups. (*p < 0.05, **p < 0.01, ***p < 0.001, *^NS^P* > 0.05, not significant). Data represented as mean ± SD.

Finally, we explored whether *AcMETTL3* and *AcMETTL14* regulate CSBV replication. First, we inhibited host *AcMETTL3* and *AcMETTL14* expression by feeding larvae dsRNA (*dsGFP*/*dsAcMETTL3*, *AcMETTL14*) and detected knockdown efficacy at multiple postprandial time points. Ultimately, we chose to collect samples 48 and 72 h after feeding dsRNA to infect CSBV (Figures 7A,C). Infections with CSBV after feeding dsRNA, and samples were collected for detection. Following *AcMETTL3* knockdown, The copies number of CSBV significantly increased compared to the control group, proving that *AcMETTL3* activity has a significant antiviral effect against CSBV replication (Figure 7B). In contrast, *AcMETTL14* has no effect on CSBV replication (Figure 7D).

## Discussion

Chinese sacbrood disease, commonly referred to as ‘honeybee cancer’ by beekeepers, has emerged as the most serious threat to *A. cerana* beekeeping. This disease causes significant population declines and substantial economic losses, posing devastating consequences for the *A. cerana* industry. The symptoms of CSBD are head turning upward, fluid sacs appearing, and the larvae becoming yellow or brown in color. At the same time, the infection of *A. mellifera* by SBV is becoming a global epidemic trend (Rueppell et al., 2020). Therefore, CSBV and SBV are serious threats to the development of beekeeping and indirectly affect growth of insect vector plants and damage ecological balance. However, minimal research has been conducted on CSBV, which severely limits the design of novel antiviral drugs for viral invasion process, resulting in the lack of effective control for CSBV.

Post-transcriptional regulation significantly influences RNA fate and function. Viral RNAs are similarly subject to such regulation, which directly controls their biological activity (Li et al., 2022). m6A modification of viral and host cell RNA enables viral infection and host immune response during viral infection (Qiu et al., 2021). The function of m6A modification in viral infection was comprehensively summarized by Williams et al. (2019). They outlined that m6A modification positively regulates the replication of RNA viruses, such as IAV, SV40, and RSV, although these differed in their mechanisms of action. However, the exact mechanism of the replication-promoting effect of m6A modification on IAV requires further investigation. In RSV, m6A modification not only promotes RSV replication but also enhances viral pathogenicity, and knocking out the m6A modification reduces RSV pathogenicity but maintains good immunogenicity, thereby providing a model for RSV vaccine development (Xue et al., 2019). In contrast, m6A modification negatively regulates RNA replication in ZIKV and HCV; however, the mechanism of negative regulation varies. Conversely, in HCV, the production of infectious viral particles is inhibited via competition with viral RNA for nucleocapsid protein.

In addition to the regulatory effects of viral m6A modifications on RNA replication, host m6A modifications can also influence viral replication. Viral infection of the host has been shown to cause disruption of host m6A-modifying demethylase ALKBH5. This enhances the m6A modification of a-ketoglutarate lactate dehydrogenase (OGDH), which decreases mRNA stability and protein expression of OGDH, and consequently causes downregulation of OGDH-itaconate signaling pathway, leading to a reconfiguration of host cell metabolism, and thereby inhibiting viral replication. Studies in ALKBH5-deficient mice have also shown that these lose their natural immune defenses against the virus (Lin and Hemenway, 2010).

For honeybees and insects in general, there is no shortage of research related to m6A modifications. For instance, as mentioned earlier, m6A modifications are crucial for caste differentiation during the larval stage of honeybees. Additionally, studies have shown that m6A modifications in rice mediate its defense responses against insect pests (Li et al., 2024). However, these studies primarily focus on insect development or indirect effects on their behavior. In contrast, our research directly investigates the interaction between a honeybee virus—CSBV—and its host. In the present study, we detected m6A modifications in CSBV-infected and healthy larvae and changes in gene expression during CSBV infection. We also applied MeRIP-seq to analyze the levels of m6A modifications in CSBV-infected *A. cerana* larvae. And demonstrated the differences in m6A methylation profiles and mRNA expression profiles between control and CSBV-infected *A. cerana* larvae. To the best of our knowledge, this is the first comprehensive high-throughput study of mRNA transcripts and m6A modifications in CSBV-infected *A. cerana* larvae.

Further research indicated m6A modifications may critically influence CSBV infection through gene expression regulation. Localization analysis of m6A modifications in mRNA revealed that most of m6A modifications occurred in CDS region between the control and CSBV-infected groups, suggesting that m6A modifications play a key role in mRNA selective splicing and translation efficiency (Lin et al., 2019). Furthermore, the m6A peak was predominantly enriched in CDS region and not in 3′-UTR region (near the stop codon), which is slightly different from the m6A enrichment observed in most model mammalian and avian mRNAs (Tao et al., 2017; Fan et al., 2019; Lin et al., 2019; Zhao et al., 2022). Our analysis of the whole CSBV genome revealed widespread presence of DR(m6A) CH-conserved motifs in CSBV genome, such as the presence of AGAGAA sequence stock between base pairs 1,424 and 1,429, 1942 and 1947, 1997 and 2002, and 7,024 and 7,029; the GGAGAA sequence was present between base pairs 1,649 and 1,654, 2,916 and 2,921, and 7,593 and 7,598; the AAAGAA sequence was present between base pairs 646 and 651, 2,184 and 2,189, and 8,218 and 822; and the AAAGAA sequence was present between base pairs 7,338 and 7,343. It is noteworthy that during our MeRIP-Seq analysis, we identified the m6A modification motifs in *A. cerana* larvae as ACCACCA and ACAUCAG, which differ from the conventional DRACH/RRACH motifs. This divergence may be attributed to the host specificity of insects. The underlying reasons for this difference and whether it influences the functional outcomes of m6A modifications in a similar manner remain unclear. Investigating these questions will be a critical focus of future research. The RNA modification of CSBV infection model was detected at nucleoside level using liquid chromatography tandem mass spectrometry (LC–MS/MS), while healthy larvae were used as a control. The results of KEGG enrichment analysis and GO analysis revealed that cell death pathways, cell metabolic pathways, and insect neurohormonal regulation may be the key host regulatory pathways affecting CSBV infection. This naturally leads us to hypothesize that certain cell death mechanisms associated with m6A modification (e.g., apoptosis, ferroptosis, etc.) may influence the susceptibility of larvae to CSBV. Additionally, insect metabolic regulation mechanisms (such as lipid metabolism, glucose metabolism, amino acid metabolism, etc.) and hormone synthesis pathways (e.g., ecdysone, juvenile hormone, etc.) could play a role in viral replication. These analytical insights provide new directions for further exploration of the pathogenic mechanisms of CSBV.

m6A modification can also effect BmNPV infection in silkworm. m6A modification could directly regulate the expression of the BmNPV structural protein VP39, suggesting that m6A modification may be a novel epigenetic mechanism regulating BmNPV infection. A new virulence strategy for the prevention of BmNPV disease (Zhang et al., 2020). Another study demonstrated that m6A modifications regulate the stability of the Hsc70 gene in the silkworm (*Bombyx mori*), thereby inhibiting subsequent viral infection (Chen et al., 2025). Another study highlights the dynamic interplay between m6A modification and viral infection. Following infection by Rice black-streaked dwarf virus (RBSDV) in the small brown planthopper (*Laodelphax striatellus*), a significant decrease in m6A modification levels was observed. The study confirmed that m6A modification restricts viral replication, while the virus in turn counteracts m6A modification to achieve persistent transmission (Tian et al., 2021). Comparing the above relationship between insect virus infection and m6A modification, we asked whether m6A modification can also reverse the replication of CSBV, and what is the underlying mechanism. Our team previously discovered an association between Chinese sacbrood virus (CSBV) infection in 4-day-old larvae and host m6A modifications. Specifically, we demonstrated that *AcMETTL3*—a key methyltransferase in bees—suppresses the expression of the immune gene *AF9*, thereby mediating immunosuppression (Bai et al., 2025). However, critical questions remain unresolved: Does *AcMETTL3* directly influence CSBV replication? And are there additional methylation-regulated genes that impact viral propagation? These questions form the central focus of the current study.

We performed qPCR analysis on two bee-encoded methylation enzymes to further examine the association between m6A modifications and CSBV infection. The expression of *AcMETTL14* did not significantly change compared with that in the control group, but the expression level of *AcMETTL3* was significantly upregulated by virus infection. Further study results showed that the virus replication level increased after infection with *AcMETTL3* compared with the control group, demonstrating the potential antiviral effect of *AcMETTL3*. The molecular mechanism by which *AcMETTL3* inhibits CSBV replication remains unclear. After identifying a correlation between host gene expression and m6A modification upon CSBV infection through MeRIP-Seq, we hypothesized whether the key methylation enzymes METTL3/14, known in mammals, might play a similar regulatory role in bees. Preliminary experiments have shown that *AcMETTL3* modulates CSBV replication, raising the question of whether it is the key molecule linking host m6A modification to CSBV infection regulation. Additionally, whether downstream regulatory genes are involved requires further validation and screening. As is well known, METTL3 typically marks target genes and mediates downstream m6A modifications to affect the stability of target genes and regulate their expression. The question then arises: does *AcMETTL3* also target and regulate key immune genes in honeybees’ major immune pathways (such as the RNAi pathway, IMD pathway, Toll pathway, autophagy pathway, and JNK pathway, etc.)? What are the effects of this targeted binding and marking on the expression of critical immune genes, and could these effects be the key reason for *AcMETTL3*’s negative regulation of CSBV? In the future, to address these questions, we will first need to perform bioinformatics analysis and prediction of *AcMETTL3* target genes, and experimentally validate their interactions. We will further investigate how *AcMETTL3* affects the stability of these target genes. Finally, by knocking down *AcMETTL3* in larvae and examining the impact of this targeted modification on entire immune pathways (at both gene and protein expression levels), we aim to elucidate the molecular mechanism by which *AcMETTL3* suppresses CSBV replication. Based on the research foundation established in this study, we will achieve more research progress in the epitranscriptomic regulation of honeybee immunity.

As a dynamic post-transcriptional RNA modification, m6A has emerged as an important focus in viral transcriptome studies. During viral infection, m6A modifications modulate virus-host interactions, directly impacting both viral replication and host antiviral responses. The growing significance of m6A modification in viral infection establishes it as a potential antiviral target. Utilizing MeRIP-Seq technology, this study provides the first evidence that CSBV infection alters m6A modification patterns in *A. cerana* larvae, while comprehensively characterizing mRNA methylation profiles. Our findings demonstrate that the m6A modification gene *AcMETTL3* negatively regulates viral replication despite its upregulation during viral infection. These results establish a foundation for investigating m6A-mediated regulation of CSBV replication and its broader roles in viral pathogenesis.

## Data Availability

The datasets presented in this study can be found in online repositories. The names of the repository/repositories and accession number(s) can be found in the article/supplementary material.

## Footnotes

^1^ http://mirrors.nju.edu.cn/bioconductor/packages/3.10/bioc/vignettes/metagene2/inst/doc/metagene2.html

^2^ http://www.geneontology.org

^3^ https://david.ncifcrf.gov

^4^ http://www.igv.org
